# The Effectiveness of Internet Cognitive Behavioural Therapy (iCBT) for Depression in Primary Care: A Quality Assurance Study

**DOI:** 10.1371/journal.pone.0057447

**Published:** 2013-02-22

**Authors:** Alishia D Williams, Gavin Andrews

**Affiliations:** 1 School of Psychiatry, University of New South Wales, Sydney, New South Wales, Australia; 2 Clinical Research Unit for Anxiety and Depression, St. Vincent's Hospital, Sydney, New South Wales, Australia; Linkoping University, Sweden

## Abstract

**Background:**

Depression is a common, recurrent, and debilitating problem and Internet delivered cognitive behaviour therapy (iCBT) could offer one solution. There are at least 25 controlled trials that demonstrate the *efficacy* of iCBT. The aim of the current paper was to evaluate the *effectiveness* of an iCBT Program in primary care that had been demonstrated to be efficacious in two randomized controlled trials (RCTs).

**Method:**

Quality assurance data from 359 patients prescribed the Sadness Program in Australia from October 2010 to November 2011 were included.

**Results:**

Intent-to-treat marginal model analyses demonstrated significant reductions in depressive symptoms (PHQ9), distress (K10), and impairment (WHODAS-II) with medium-large effect sizes (Cohen's *d* = .51–1.13.), even in severe and/or suicidal patients (Cohen's *d* = .50–1.49.) Secondary analyses on patients who completed all 6 lessons showed levels of clinically significant change as indexed by established criteria for remission, recovery, and reliable change.

**Conclusions:**

The Sadness Program is effective when prescribed by primary care practitioners and is consistent with a cost-effective stepped-care framework.

## Introduction

Depression is a common, recurrent, and debilitating problem [1]. Although evidence-based treatments exist, most people with depression do not obtain such treatment [2]. Of those who do access evidence-based treatments, approximately 25% do not improve [3]. Most adults who seek help for depression are treated in primary care settings where under-recognition remains high [4] and treatment may not be ideal [5]. Integration of mental health specialists into primary care sites has resulted in better treatment outcomes [6], but pragmatic and financial reasons are likely to preclude complete adoption of this model. One cost-effective and pragmatic means of increasing the quality of treatment available in primary care settings is through the use of internet-based cognitive behavioural therapy (iCBT) programs. Internet-based treatment affords many benefits over the traditional face-to-face modality, such as high fidelity, greater accessibility, convenience, and reduced cost to patients [7]. In a systematic review of 25 controlled trials (total n = 5,719) of iCBT for depression vs treatment as usual, placebo or wait list control [8] effect size superiority over the control groups ranged from zero to 1.18. Meta-analyses of RCTs of iCBT for depression have demonstrated moderate effect sizes that provide evidence that iCBT can be comparable to best-practice face-to-face CBT [7], [9], [10]. The CRUfAD Sadness Program (www.thiswayup.org.au/clinic) has been evaluated in a number of trials [11], [12], [13]. In a RCT comparing clinician-assisted to technician-assisted implementation of the Sadness Program, Cohen's *d* effect size superiority over the wait list control group at the end of therapy (PHQ9 scores) was 1.27, and this result was maintained at follow-up. Patient samples in research studies may be unrepresentative of those seen in primary care, but the highly structured and standardized nature of the iCBT Sadness program ensures that it can be transferred to routine practice in primary care without compromising treatment fidelity or efficacy. Whether effectiveness in practice parallels efficacy in RCTs is the core question addressed in the current paper by examining the progress of patients treated with the Sadness program in primary care. We evaluated this by examining the progress of patients treated with the Sadness program in primary care. In most efficacy studies, individuals with severe depressive symptoms and suicidal ideation are excluded from participation and are referred for traditional face-to-face treatment. Therefore systematic evidence that iCBT is appropriate for this population is lacking. We advise prescribing clinicians to exclude patients who are very severe or who are actively suicidal, but it is unknown to what extent clinicians adhere to this guidance. The current quality assurance study sought to quantify the proportion of individuals enrolled in the Sadness Program who were either severely depressed and/or expressing suicidal ideation and secondly, to determine whether treatment was effective for this group of patients.

## Methods

### Ethics Statement

The current paper was written as part of the Quality Assurance activities of St. Vincent's Hospital. At St. Vincent's Hospital the Human Research Ethics Committee does not consider clinical audits. That responsibility is vested with the Quality Assurance and Patient Safety Unit with whom a copy of this paper was lodged prior to submission. The Quality Assurance and Patient Safety Unit does not formally approve research projects, but assesses submitted reports for adherence to the Clinical Governance framework guidelines. The current quality assurance study adhered to these guidelines by examining the type of patient being prescribed the Sadness Program in primary care and reports on the effectiveness of the program. Data was necessarily confined to measures used as a routine to inform practitioners about the progress of their patients. All patients provided electronic informed consent that their pooled data could be used for quality assurance purposes.

### The Sadness Program

The Sadness Program has been described in detail previously [11], [12]. Patients were provided with a prescription from a GP or clinician registered with CRUfAD in order to enrol in the Sadness Program. As routine practice, prescribing clinicians were advised that patients are unlikely to benefit if they have very severe depression, persistent suicidal thoughts, drug or alcohol dependence, schizophrenia, bipolar disorder, or are taking atypical antipsychotics or benzodiazepines. Clinical responsibility was maintained by the prescribing clinician who received automatic updates via email regarding each patient's progress. The prescribing clinician also received an email alert if a patient's scores on the Kessler-10 (K10) Psychological Distress Scale indicated elevated distress or the patient endorsed suicidality on the Patient Health Questionnaire (PHQ-9). The Sadness Program was developed so that a patient cannot advance to the subsequent lesson without first completing the preceding lesson, downloading the associated homework components, and then waiting 5 days (to ensure sufficient time to review the materials and to complete the homework tasks). All patients have 10 weeks to complete the program and are encouraged to progress through each lesson at a pace of 1 lesson per every 1–2 weeks. Patient progress is tracked automatically through the CRUfAD Clinic system. The program consists of six online lessons representing best practice CBT as well as regular homework assignments and access to supplementary resources. Each lesson was designed using a cartoon narrative and included: psycho-education, behavioural activation, cognitive restructuring, graded exposure, problem solving, assertiveness skills, and relapse prevention.

### Participants

Data were collected from 359 patients with a prescription for the Sadness Program from October 2010 to November 2011. The mean age of patients was 41.59 (*SD* = 14.15) and 59% were female. Fifty-four percent of patients were from an Australian rural or remote community (defined based on the geographical location of the prescribing clinician's practice). For secondary analyses, patients were classified into three groups based on the number of lessons completed. Patients who completed all six lessons are referred to as Completers. Non-Completers were separated into two groups based on evidence that the treatment dose curve peaks at lesson 4 [14], therefore patients who completed at least 4 lessons are referred to as Non-Completers and patients who completed between 1–3 lessons are referred to as Drop-Outs.

### Measures

#### Patient Health Questionnaire

(PHQ-9; [15]). The PHQ-9 is a self-report questionnaire corresponding to the DSM-IV criteria for major depressive disorder. Each item is rated in frequency on a 4-point (0 = not at all, 3 = nearly every day) scale. Total scores range from 0 to 27 with higher scores reflecting higher levels of psychopathology. Depression severity categories correspond to the following scores: 0–9 = normal, 10–14 = mild, 15–19 = moderate, 20–23 = severe, 24–27 = very severe). A PHQ-9 score of ≥10 is used as a clinical cut-off for probable DSM-IV diagnosis of MDD [16]. The PHQ-9 demonstrates good psychometric properties and has been used extensively to measure treatment outcomes during internet CBT interventions targeting depression and anxiety [17], [18].

#### Kessler-10 (K10) Psychological Distress Scale

[19]. The K10 consists of 10 items ranked on a five point scale designed to measure non-specific psychological distress. The K10 is completed prior to each lesson a**s** a means of tracking patient distress. If a patient endorses high K10 scores (>35) or evidences an increase by more than 0.5 standard deviations, an automatic alert is emailed to the prescribing clinician. The K10 possesses strong psychometric properties [14], [19], [20].

#### World Health Organization Disability Assessment Schedule-II

(WHODAS-II). The WHODAS-II contains 12 items designed to measure disability and activity limitation in the past 30 days in a variety of domains: 1) understanding and communicating, 2) self-care, 3) mobility, 4) interpersonal relationships, 5) work and household roles, and 6) community roles. Each of these domains loads significantly onto one underlying latent factor of global disability [21]. Scores range from 0 to 60, with higher scores indicating greater disability. The WHODAS-II demonstrates strong psychometric properties [22].

### Statistical Analyses

Intent-to-treat (ITT) marginal model analyses were used to measures the change in outcome measures across time in the full sample (including drop-outs). This method accounts for missing data due to participant drop-out without assuming that the last measurement was stable (the last observation carried forward assumption- LOCF; [23]) and is appropriate for pre-post only designs [24]. Effects were modelled using the restricted maximum likelihood (REML) model estimation method with an autoregressive (AR1) covariance structure specified to account for the correlation between the measures at each time point. Simulation studies have demonstrated the superiority of complete case analysis over LOCF methods in pre-post designs [24], [25]. For secondary analyses of Completers and Non-Completers, ANOVA and χ^2^ were conducted. The groups were compared on a range of variables including: age, sex, prescribing clinician's profession, rurality (yes, no), and pre-course mean scores on the PHQ9, K10, and WHODAS-II. Cohen's *d* within-group effect sizes were computed based on the pooled standard deviation, and corrected for repeated-measurements. Clinically significant change was calculated in Completers only. Clinically significant change was defined in three ways. *Remission* was defined as a post-treatment score below the optimal cut-score for a probable diagnosis of depression on the PHQ9 in patients who initially scored above threshold (>9). *Recovery* was defined as a reduction of at least 50% of pre-treatment PHQ9 scores. *Reliable improvement* was defined as a decrease in at least 5 points on the PHQ9 and a change in depression severity category based on PHQ9 cut-scores (i.e., from moderate to mild) from pre-to-post treatment based on the recommendations of [26]. All analyses were conducted using SPSS version 20.

## Results

### Sample Characteristics

Of the 359 patients initially enrolled, 26.5% endorsed PHQ9 scores within the 0–9 subthreshold range, 26% were classified as mild, 23% as moderate, 17% as severe, and 7.5% as very severe. One-hundred and ninety four (54%) patients completed all 6 lessons of the program (Completers), 113 (32%) completed at least 4 lessons (Non-completers), and 52 (15%) completed between 1 and 3 lessons (Drop-outs). Program completion was unrelated to a number of variables, including patient gender, χ^2^ (2) = .49, *p*>.05, the health-care profession of the prescriber (psychologist, GP, other health practitioner), χ^2^ (4) = 1.61, *p*>.05, and whether the patient lived in a rural area or not, χ^2^ (2) = 2.32, *p*>.05. Program completion did, however, vary as a function of age, *F* (2, 356) = 7.08, *p* = .001, *η* ^2^ = .04. Waller-Duncan post-hoc testing (adjusting for unequal n's) revealed that Completers were significantly older (*M* = 44.13, *SD* = 13.90) than Non-Completers (*M* = 39.00, *SD* = 14.30) and Drop-Outs (*M* = 38.42, *SD* = 13.74). Age did not differ significantly between the Non-Completers and Drop-outs, *p*>.05. In order to gain a better understanding of variables related to treatment drop-out, patient's baseline scores on the primary measures were also compared. There were no differences between scores on the PHQ9, *F* (2, 356) = .68, *p*>.05, on the K10, *F* (2, 356) = 2.24, *p*>.05, or on the WHODAS-II, *F* (2, 356) = 2.70, *p*>.05. There was also no significant difference in the amount of time each group spent on the first lesson of the program, *F* (2, 356) = 2.07, *p*>.05. These variables were also entered as predictors in a multivariate logistic regression model predicting completion status (0 = completed<6 lessons, 1 = completed 6 lessons). The only significant predictor was age (*p*<.001), indicating that each additional year in age was associated with a 1.03 increase in the likelihood of completing all 6 lessons.

### Change in Primary Outcome Measures

Marginal model analyses were conducted in the full sample (ITT) on the primary outcome measures. There was a main effect of time for PHQ9 scores, *F* (1, 225.91) = 269.56, *p*<.001, for K10 scores, *F* (1, 233.33) = 376.39, *p*<.001, and for WHODAS-II scores, *F* (1, 226.11) = 84.82, *p*<.001, indicating a significant reduction in all three measures from baseline to completion of the program. Within-group effect sizes, calculated using the observed means and the pooled standard deviation (and correcting for repeated-measures), indicated medium-large effects for all primary outcome measures (see Table 1). These analyses were repeated in the sample meeting threshold criteria for a probable diagnosis of depression (PHQ9>10) with all main effects remaining significant.

**Table 1:** Observed means (standard deviations) for primary measures and within-group effect sizes (Cohen's *d*).

Group	Lesson 1		Lesson 6								
Full Sample	M	SD	M	SD	Mean Difference	(df)	*F*	*R* (for within-group ES)	Effect Size Cohen's *d*		(95% CI)
PHQ9	14.08	6.04	8.12	6.12	5.96	(1, 236.66)	265.45**	.59	.97		.77–1.16
K10	29.40	7.15	20.22	8.75	9.18	(1, 233.33)	376.39**	.63	1.13		.93–1.32
WHODAS	17.48	8.45	13.02	8.93	4.46	(1, 226.11)	84.82**	.68	.51		.31–.70
Suicidal Ideation											
PHQ9	17.11	5.44	10.71	6.46	6.40	(1, 115.75)	144.29**	.51	1.08		.80–1.35
K10	32.84	6.46	24.04	9.14	8.80	(1, 136.34)	173.99**	.60	1.07		.79–1.34
WHODAS	20.94	8.47	16.64	8.81	4.30	(1, 125.72)	31.30**	.61	.50		.22–.77
PHQ9-Suicide Item	1.58	.75	.70	.83	.88	(1, 245.05)	55.39*	.23	1.10		.82–1.37
Severe Depression & Suicidal Ideation											
PHQ9	23.11	2.16	14.71	6.71	8.40	(1, 70.84)	111.01**	.40	1.49		1.01–1.96
K10	37.71	4.77	28.51	9.91	9.20	(1, 60.35)	66.93**	.61	1.02		.54–1.49
WHODAS	26.63	6.60	21.49	8.39	5.14	(1, 54.35)	13.14*	.39	.67		.20–1.13
PHQ9-Suicide Item	1.97	.82	.97	.95	1.00	(1, 51.31)	44.93**	.45	1.12		.64–1.59

Note. PHQ9  =  Patient Health Questionnaire -9 item; K10  =  Kessler Distress Scale – 10 item; WHODAS  =  World Health Organization Disability Assessment Schedule-II; PHQ9 –Suicide  =  Patient Health Questionnaire – item 9; *r*  =  Pearson correlation between Lesson 1 & Lesson 6 scores; **p<.001, *p = .001.

### Treatment Effectiveness for Severe and Suicidal Patients

Approximately one-quarter of patients prescribed the Sadness course had PHQ9 scores in the severe range (17% severe, 8% very severe). Additionally, 31% (n = 112) endorsed suicidal thoughts several days during the 2-week time period prior to commencing the program, 13% (n = 46) endorsed suicidal ideation for more than half the days, and 9% (n = 31) endorsed suicidal thoughts nearly every day during this time-frame. Of the patients endorsing suicidal ideation, the majority (53%) completed all 6 lessons (68% completed at least 4 lessons), suggesting that the presence of suicidal ideation was not a barrier to treatment adherence. To determine whether suicidal ideation was a barrier to treatment response (irrespective of baseline depression severity), marginal model analyses and Cohen's *d* within-group effect sizes were calculated in patients who endorsed suicidal thoughts at baseline (n = 189). Results are reported in Table 1. All main effects were significant, all *p*'s≤.001, with mean reductions corresponding to medium-large effect sizes. These analyses were repeated in severe patients (PHQ9≥20) who endorsed suicidal ideation (n = 72). All main effects were significant, all *p*'s≤.001, with mean reductions corresponding to medium-large effect sizes.

### Clinically Significant Change in Completers

Of those patients who met threshold criteria at baseline for a probable diagnosis of depression (PHQ9>9) and who completed all 6 lessons, 63% (91/144) evidenced remission (PHQ9<9). Forty-nine percent (71/144) evidenced recovery (at least a 50% reduction in baseline PHQ9 scores). For clinically reliable change, 54% (n = 77/144) evidenced a reduction of at least 5 points on the PHQ9 in combination with a change in depression severity category from higher to lower. Clinically reliable change was unrelated to the profession of the prescribing clinician, χ^2^ (4) = 4.08, *p*>.05.

### Treatment Response in Completers, Non-Completers and Drop-Outs

As the K10 was administered prior to each lesson, data were available to calculate reductions in distress as a function of each lesson completed irrespective of program completion. A significant reduction in distress was defined as at least a 1 *SD* (7.5) decrease in K10 scores from lesson 1 to the final lesson completed. This was a conservative definition as decreases of 7 points have been shown to correspond to reliable improvement [27]. Table 2 reports the proportion of patients who demonstrated a significant reduction based on this criterion. Consistent with previous findings that the greatest reduction in distress scores occurs within the first four lessons of the Sadness Program [14], [28], nearly half (44%) of patients who dropped out after lesson 4 appeared to have benefitted from the program.

**Table 2:** Mean reductions in K10 scores for Drop-Outs, Non-Completers, and Completers by lesson.

	Lesson 2 Drop-Out	Lesson 3 Drop-Out	Lesson 4 Non-Completer	Lesson 5 Non-Completer	Lesson 6 Completer
**n**	30	36	25	27	194
**Mean K10 Reduction** **(SD)**	2.03 (5.70)	4.00 (5.13)	5.72 (5.31)	8.29 (8.77)	9.21 (7.00)
**% Significant Reduction**	13%	22%	44%	33%	60%

Note.;% significant reduction  =  proportion of Drop-Outs who demonstrated at least 1 SD reduction from lesson 1 K10 scores.

## Discussion

The current study provides evidence of the effectiveness of an internet-based CBT (iCBT) program for depression, the Sadness Program, prescribed by primary-care practitioners. Findings indicate that the Sadness Program is effective in reducing depressive symptoms, distress, and impairment with corresponding medium-large effect sizes in primary outcome measures. Approximately 55% of patients who completed all 6 lessons of the program evidenced clinically significant change as indexed by established criteria for remission, recovery, and reliable change.

There have been mixed findings regarding the effectiveness of standard CBT in treatment of severe depression [29]. In the current study, one-quarter (25%) of patients were in the severe-very severe range for depression symptoms. The results of the current study suggest that the Sadness Program is effective in reducing depressive symptoms, distress, and impairment in a large proportion of patients presenting with symptoms in the severe range. The current findings also suggest that the presence of suicidal ideation is not a barrier to treatment adherence or response as patients with active suicidal thoughts demonstrated significant reductions in all primary outcome variables with corresponding medium-large effect sizes. The population attributable ratio (PAR) for depression in suicidal behaviour has been estimated at 80 percent, meaning that 80% of suicidal behaviour would be eradicated if depression did not occur [30], therefore the current findings of reduced depressive symptoms and suicidal ideation are not trivial. The Sadness Program is designed to automatically alert the prescribing clinician (via email) of suicidal ideation or increased patient distress; however it remains the responsibility of the prescribing clinician to act on these alerts. It is also important to identify patients who are not responding given evidence that recurrence is partially influenced by symptom reduction during treatment. Research has demonstrated that individuals who were asymptomatic at follow-up had much lower rates of recurrence than treated individuals with residual symptoms at follow-up (66% vs 87%, respectively) [31].

Conversely, a consequence of under-recognition of subthreshold or mild depression is that effective early interventions strategies, that may otherwise reduce the risk of patients becoming fully symptomatic, are not being optimally used [32]. It is interesting to note that nearly 27% of patients prescribed the Sadness Program did not meet threshold criteria for a probable diagnosis of depression. It is unknown whether these patients were in fact subthreshold, were patients in remission from a previous episode, or were patients who were simply mis-prescribed the program. It would appear that the program material was relevant to the majority of these patients so it is unlikely that patients were incorrectly identified. The decision to retain these subthreshold cases in the current analyses was made on the basis that the primary aim of the current study was to evaluate the effectiveness of the Sadness Program *as it is prescribed in routine practice.* We presume that these patients were either experiencing chronic mild depression or were patients with a history of recurrent depression who were currently in remission and may have been interested in learning how to better manage their symptoms. Inclusion of routine questions regarding depression history may be a valuable addition to the Sadness Program.

Program adherence (54%) in the current study was lower than reported in two previous RCTs investigating the efficacy of the Sadness Program [11], [12], 74% and 75%, respectively, but was consistent with median completion rates (56%) reported in a meta-analysis of computerized CBT treatments [33]. The only variable related to program completion was age: Completers were significantly older than Non-Completers and Drop-Outs. Research is required to assess variables that may interfere with treatment commitment, adherence, and response. It is important to note that a large proportion of patients evidenced a significant reduction in global distress prior to drop-out, suggesting benefit despite program non-completion. Future research could determine whether reducing the length of the program leads to better adherence rates.

The current findings need to be considered in light of a number of limitations. As data was collected in the context of routine clinical practice there was no comparison group, hence the effects of natural remission or placebo response over the 10 weeks cannot be separated from response to the specific treatment. It is also important to note that we did not collect information on medication use. It is likely that a proportion of patients presenting to GPs would have also been prescribed a course of antidepressants; however clinically significant treatment response did not vary as a function of the clinician being licensed to prescribe medication. Further, the within-group effect size for change on the PHQ9 was not larger than that obtained in the RCTs of the Sadness Program (where patients who had commenced or altered their antidepressant medication within the last month were excluded). Data were collected to ensure ongoing quality assurance. A benefit of this type of data is that it provides an indication of treatment effectiveness in absence of confounding variables that may indirectly influence treatment response. Had data been collected in the context of a field RCT, the motivation of primary care clinicians to adhere to the Sadness Program guidelines may have increased as a function of external evaluation. Furthermore, the use of no treatment or wait-list control comparisons once efficacious treatments have been identified is increasingly raising ethical questions [34], particularly in the context of primary care. Effectiveness research is aimed at evaluating the feasibility, acceptability, and effectiveness of treatments in environments where most patients will be treated [35]. We are confident that the current results reflect routine practice and therefore can be generalized to other primary care settings.

In conclusion, requisite conditions for improving treatment of depression in primary care have been identified and include organized treatment programs, monitoring of treatment adherence, and guidance from mental health specialists as educators and consultants [36]. The Sadness Program, and other iCBT treatments (including CRUfAD's GAD Program [37]) represents a cost-effective means of ensuring these criteria are met. The current paper provides preliminary evidence of the effectiveness of this form of treatment delivery in primary care and is consistent with a stepped-care framework identified in Australian and NICE guidelines as the method by which treatments for depression, and other disorders, should be delivered [38], [39].

## References

[1] Andrews G (2001) Should depression be managed as a chronic disease? BMJ: British Medical Journal 322: 419–421. 11179166 10.1136/bmj.322.7283.419 PMC1119639

[2] Kessler RC, Berglund P, Demler O, Jin R, Koretz D, et al (2003) The epidemiology of major depressive disorder: Results from the National Comorbidity Survey Replication (NCS-R). JAMA: Journal of the American Medical Association 289: 3095–3105. 12813115 10.1001/jama.289.23.3095

[3] DeRubeis RJ, Hollon SD, Amsterdam JD, Shelton RC, Young PR, et al (2005) Cognitive Therapy vs Medications in the Treatment of Moderate to Severe Depression. Archives of general psychiatry 62: 409–416. 15809408 10.1001/archpsyc.62.4.409

[4] Mitchell AJ, Vaze A, Rao S (2009) Clinical diagnosis of depression in primary care: A meta-analysis. The Lancet 374: 609–619. 10.1016/S0140-6736(09)60879-5 19640579

[5] Wolf NJ, Hopko DR (2008) Psychosocial and pharmacological interventions for depressed adults in primary care: A critical review. Clinical psychology review 28: 131–161. 17555857 10.1016/j.cpr.2007.04.004

[6] Katon W, Von Korff M, Lin E, Walker E, Simon GE, et al (1995) Collaborative management to achieve treatment guidelines. Impact on depression in primary care. JAMA: the journal of the American Medical Association 273: 1026–1031. 7897786

[7] Andersson G, Cuijpers P (2009) Internet-based and other computerized psychological treatments for adult depression: A meta-analysis. Cognitive behaviour therapy 38: 196–205. 20183695 10.1080/16506070903318960

[8] Johansson R, Andersson G (2012) Internet-based psychological treatments for depression. Expert Review of Neurotherapeutics 12: 861–870. 22853793 10.1586/ern.12.63

[9] Cuijpers P, Donker T, Johansson R, Mohr DC, van Straten A, et al (2011) Self-guided psychological treatment for depressive symptoms: A meta-analysis. PloS one 6. 10.1371/journal.pone.0021274 PMC3119687 21712998

[10] Andrews G, Cuijpers P, Craske MG, McEvoy P, Titov N (2010) Computer therapy for the anxiety and depressive disorders is effective, acceptable and practical health care: A meta-analysis. PloS one 5. 10.1371/journal.pone.0013196 PMC2954140 20967242

[11] Titov N, Andrews G, Davies M, McIntyre K, Robinson E, et al (2010) Internet treatment for depression: a randomized controlled trial comparing clinician vs. technician assistance. PLoS One 5: e10939. 20544030 10.1371/journal.pone.0010939 PMC2882336

[12] Perini S, Titov N, Andrews G (2009) Clinician-assisted Internet-based treatment is effective for depression: randomized controlled trial. The Australian and New Zealand journal of psychiatry 43: 571–578. 19440890 10.1080/00048670902873722

[13] Perini S, Titov N, Andrews G (2008) The climate sadness program of internet-based treatment for depression: A pilot study. E-Journal of Applied Psychology 4: 18–24.

[14] Sunderland M, Wong N, Hilvert-Bruce Z, Andrews G (2012) Investigating trajectories of change in psychological distress amongst patients with depression and generalised anxiety disorder treated with internet cognitive behavioural therapy. Behaviour Research and Therapy 50: 374–380. 22498311 10.1016/j.brat.2012.03.005

[15] Kroenke K, Spitzer RL, Williams JB (2001) The PHQ-9: validity of a brief depression severity measure. Journal of General Internal Medicine 16: 606–613. 11556941 10.1046/j.1525-1497.2001.016009606.x PMC1495268

[16] Zuithoff NP, Vergouwe Y, King M, Nazareth I, van Wezep MJ, et al (2010) The Patient Health Questionnaire-9 for detection of major depressive disorder in primary care: consequences of current thresholds in a crosssectional study. BMC family practice 11: 98. 21144018 10.1186/1471-2296-11-98 PMC3009622

[17] Titov N, Dear BF, Schwencke G, Andrews G, Johnston L, et al (2011) Transdiagnostic internet treatment for anxiety and depression: a randomised controlled trial. Behaviour Research and Therapy 49: 441–452. 21679925 10.1016/j.brat.2011.03.007

[18] Dear BF, Titov N, Schwencke G, Andrews G, Johnston L, et al (2011) An open trial of a brief transdiagnostic internet treatment for anxiety and depression. Behaviour Research and Therapy 49: 830–837. 21993153 10.1016/j.brat.2011.09.007

[19] Kessler RC, Andrews G, Colpe LJ, Hiripi E, Mroczek DK, et al (2002) Short screening scales to monitor population prevalences and trends in non-specific psychological distress. Psychological medicine 32: 959–976. 12214795 10.1017/s0033291702006074

[20] Andrews G, Slade T (2001) Interpreting scores on the Kessler Psychological Distress Scale (K10). Australian New Zealand Journal of Public Health 25: 494–497. 11824981 10.1111/j.1467-842x.2001.tb00310.x

[21] Andrews G, Kemp A, Sunderland M, Von Korff M, Ustun TB (2009) Normative data for the 12 item WHO Disability Assessment Schedule 2.0. PLoS One 4: e8343. 20020047 10.1371/journal.pone.0008343 PMC2791224

[22] Federici S, Meloni F, Lo Presti A (2009) International literature review on WHODAS II (World Health Organization Disability Assessment Schedule II). Life Span and Disability 12: 83–110.

[23] Gueorguieva R, Krystal JH (2004) Move Over ANOVA Progress in Analyzing Repeated-Measures Data and Its Reflection in Papers Published in the Archives of General Psychiatry. Archives of General Psychiatry 61: 310–317. 14993119 10.1001/archpsyc.61.3.310

[24] Salim A, Mackinnon A, Christensen H, Griffiths K (2008) Comparison of data analysis strategies for intent-to-treat analysis in pre-test-post-test designs with substantial dropout rates. Psychiatry Research 160: 335–345. 18718673 10.1016/j.psychres.2007.08.005

[25] Salim A, Mackinnon A, Christensen H, Griffiths K (2008) Comparison of data analysis strategies for intent-to-treat analysis in pre-test–post-test designs with substantial dropout rates. Psychiatry research 160: 335–345. 18718673 10.1016/j.psychres.2007.08.005

[26] McMillan D, Gilbody S, Richards D (2010) Defining successful treatment outcome in depression using the PHQ-9: A comparison of methods. Journal of Affective Disorders 127: 122–129. 20569992 10.1016/j.jad.2010.04.030

[27] Gonda T, Deane FP, Murugesan G (2012) Predicting clinically significant change in an inpatient program for people with severe mental illness. The Australian and New Zealand journal of psychiatry 46: 651–658. 22528976 10.1177/0004867412445527

[28] Hilvert-Bruce Z, Rossouw P, Wong N, Sunderland M, Andrews G (2012) Adherence as a determinant of effectiveness of internet cognitive behavioural therapy for anxiety and depressive disorders. Behaviour Research and Therapy 50: 463–468. 22659155 10.1016/j.brat.2012.04.001

[29] Kessler D, Lewis G, Kaur S, Wiles N, King M, et al (2009) Therapist-delivered internet psychotherapy for depression in primary care: A randomised controlled trial. The Lancet 374: 628–634. 10.1016/S0140-6736(09)61257-5 19700005

[30] Beautrais AL, Joyce PR, Mulder RT, Fergusson DM, Deavoll BJ, et al (1996) Prevalence and comorbidity of mental disorders in persons making serious suicide attempts: a case-control study. The American journal of psychiatry 153: 1009–1014. 8678168 10.1176/ajp.153.8.1009

[31] Mueller TI, Leon AC, Keller MB, Solomon DA, Endicott J, et al (1999) Recurrence after recovery from major depressive disorder during 15 years of observational follow-up. The American journal of psychiatry 156: 1000–1006. 10401442 10.1176/ajp.156.7.1000

[32] Conradi HJ, de Jonge P, Kluiter H, Smit A, van der Meer K, et al (2007) Enhanced treatment for depression in primary care: Long-term outcomes of a psycho-educational prevention program alone and enriched with psychiatric consultation or cognitive behavioral therapy. Psychological Medicine: A Journal of Research in Psychiatry and the Allied Sciences 37: 849–862. 10.1017/S0033291706009809 17376257

[33] Waller R, Gilbody S (2010) Barriers to the uptake of computerized cognitive behavioural therapy: a systematic review of the quantitative and qualitative evidence. Psychological medicine 39: 705. 10.1017/S0033291708004224 18812006

[34] Devilly GJ, McFarlane AC (2009) When Wait Lists Are not Feasible, Nothing Is a Thing That Does not Need to Be Done. [Article]: Journal of Consulting & Clinical Psychology December 2009 77(6): 1159–1168. 10.1037/a0016878 19968391

[35] Roy-Byrne PP, Sherbourne CD, Craske MG, Stein MB, Katon W, et al (2003) Moving treatment research from clinical trials to the real world. Psychiatric Services 54: 327–332. 12610239 10.1176/appi.ps.54.3.327

[36] Schulberg HC, Katon W, Simon GE, Rush A (1998) Treating major depression in primary care practice: An update of the Agency for Health Care Policy and Research practice guidelines. Archives of general psychiatry 55: 1121–1127. 9862556 10.1001/archpsyc.55.12.1121

[37] Mewton L, Wong N, Andrews G (2012) The effectiveness of computerised cognitive behavioural therapy for generalised anxiety disorder in clinical practice. Depression and Anxiety 29: 843–849. 22949296 10.1002/da.21995

[38] NICE (2009) Depression: Management of depression in primary and secondary care.: National Institute for Health and Clinical Excellence, London.

[39] Andrews G, Team T (2007) Tolkien II: a needs based, costed, stepped-care model for Mental Health Services. Sydney: World Health Organization Collaborating Centre for Classification in Mental Health.

